# A carbohydrate-grafted nanovesicle with activatable optical and acoustic contrasts for dual modality high performance tumor imaging†
†Electronic supplementary information (ESI) available: Experimental procedures. See DOI: 10.1039/c4sc03641g



**DOI:** 10.1039/c4sc03641g

**Published:** 2014-12-22

**Authors:** Xuanjun Wu, Bijuan Lin, Mingzhu Yu, Liu Yang, Jiahuai Han, Shoufa Han

**Affiliations:** a State Key Laboratory for Physical Chemistry of Solid Surfaces , The Key Laboratory for Chemical Biology of Fujian Province , The MOE Key Laboratory of Spectrochemical Analysis & Instrumentation , Innovation Center for Cell Biology, and Department of Chemical Biology , College of Chemistry and Chemical Engineering Xiamen University , Xiamen , 361005 , China . Email: shoufa@xmu.edu.cn ; Tel: +86-0592-2181728; b State Key Laboratory of Cellular Stress Biology , Innovation Center for Cell Biology , School of Life Sciences , Xiamen University , Xiamen , 361005 , China

## Abstract

Activatable molecular systems enabling precise tumor localization are valuable for complete tumor resection. Herein, we report sialic acid-capped polymeric nanovesicles encapsulating the near infrared profluorophore (pNIR@P@SA) for lysosome activation based dual modality tumor imaging. The probe features surface-anchored sialic acid for tumor targeting and a core of near infrared profluorophore (pNIR) which undergoes lysosomal acidity triggered isomerization to give optical and optoacoustic signals upon cell internalization. Imaging studies reveal high-efficiency uptake and signal activation of pNIR@P@SA in subcutaneous tumors and millimeter-sized liver tumor foci in mice. The high tumor-to-healthy organ signal contrasts and discernment of tiny liver tumors from normal liver tissues validate the potential of pNIR@P@SA for high performance optical and optoacoustic imaging guided tumor resection.

## Abstract 2

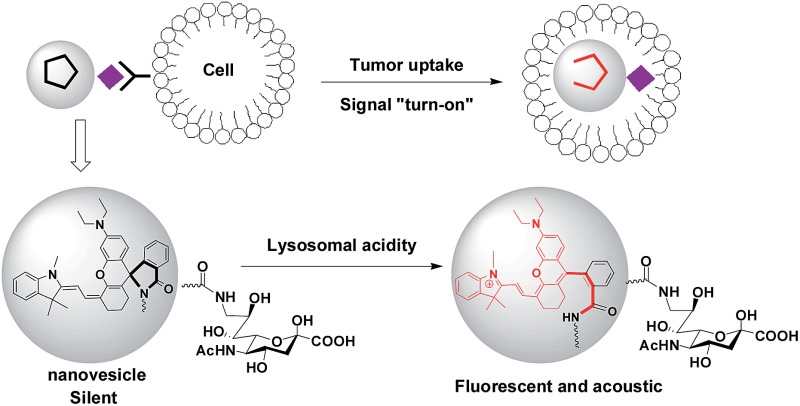
High-performance illumination of subcutaneous tumor and liver tumor foci was achieved with sialic acid-targeted acid-responsive nanovesicles which become fluorescent and photoacoustic upon internalization into tumor lysosomes.

## Introduction

With increasing cancer mortality, technologies that could improve the outcome of cancer treatment are of clinical interest. Widely employed for tumor treatment, surgical resection is often hampered by limited visibility of tiny or embedded tumors, leading to incomplete surgical ablation and ensuing tumor recurrence. As such, optical systems capable of guiding surgeons to evasive tumors are being vigorously explored.^1^ Conventional dyes lack the specificity to recognize tumor cells. To achieve high tumor-to-healthy tissue signal contrast, dyes are often armed with tumor-targeting entities which are largely confined to antibodies, folate, peptides, and aptamers, *etc.* ^2^ Sialic acids (SAs), a family of 9-carbon monosaccharides derived from *N*-acetyl-neuraminic acid, are typically located at termini of cell surface glycans.^3^ Cell surface hypersialylation is a characteristic of many cancers and the hypoxic core of solid tumors,^4^ suggesting elevated metabolic demand of SA by these tumor cells. Recently, dye-labelled SA was demonstrated for tumor detection in mice, showing the applicability of SA for *in vivo* tumor targeting.^5^

**Fig. 1:**
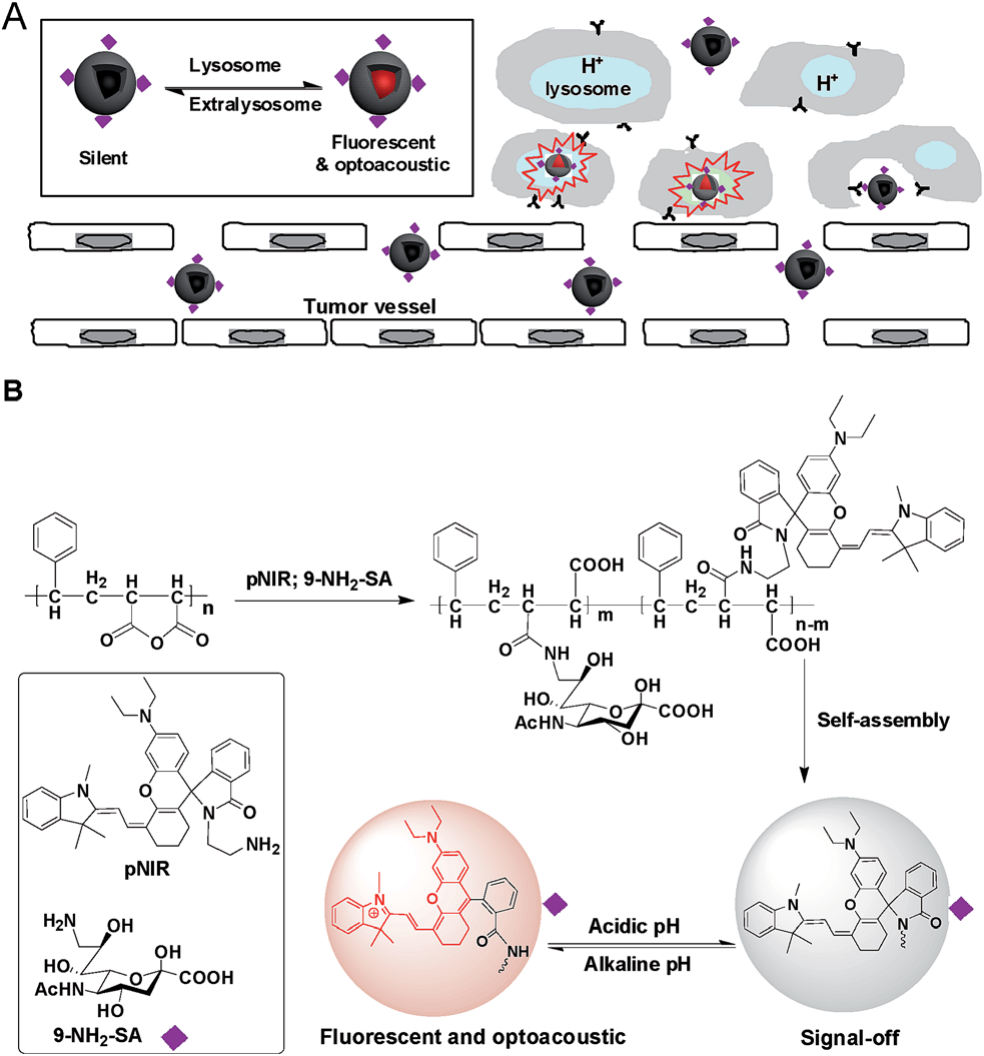
Schematic of lysosomal acidity activation based fluorescence and optoacoustic tumor imaging with pNIR@P@SA (A). The polymeric vesicle features SA anchored onto the surface at C-9 for tumor targeting and encapsulated pNIR poised for proton mediated isomerization to give NIR fluorescence and optoacoustic signals (B).

Optical systems that are activated to fluorescence-on states while remaining silent at off-target settings are advantageous for high signal-to-background contrast.^1^ Fluorescence imaging suffers strong photon diffusion in tissues whereas optoacoustic imaging employs weakly scattered ultrasound and thus enables deep tissue imaging.^6^ Recently two molecular systems with an inert reference photoacoustic signal and other variable optoacoustic signals responsive to MMP-2 enzyme or reactive oxygen species have been constructed for activatable photoacoustic imaging.^7^ Complementary to these approaches, we herein report “turn-on” imaging based on isomerization of a non-optoacoustic molecular entity into an optoacoustic agent within acidic lysosomes. To integrate the advantages of NIR fluorescence imaging (low background signals) and acoustic imaging (deep tissue penetration), we herein report a SA-capped polymersome featuring a NIR profluorophore (pNIR) for lysosome activation based optical and optoacoustic tumor imaging (Fig. 1). pNIR@P@SA comprises SA displayed on the surface of polymeric vesicles for tumor targeting, a shell of biocompatible poly[styrene-*alter*-(maleic acid)], and a hydrophobic core of pNIR responsive to lysosomal acidity. Imaging studies in tumor-bearing mice intravenously injected with pNIR@P@SA reveal “turn-on” NIR fluorescence and acoustic signals in tumors and pharmacokinetics advantageous for imaging guided surgery.

## Results and discussion

### Construction and characterization of pNIR@P@SA

Over sialylation of cell surface glycoconjugates is a hallmark of a number of cancer types and the hypoxic cores of solid tumors.^4^ Cell surface SA is metabolically attached to preceding glycan acceptors by glycosidic bonds at C-2, mostly α-2,3/6 linkages.^3^ Nanomedicine with targeting and therapeutic/imaging entities on a single particle has attracted enormous interest. Being ligands of endogenous SA-binding lectins, sialosides with C-2 glycosidic bonds have been integrated with various nanocarriers for biomedical applications.^8^ The reported tumor imaging with fluorescein isothiocyanate-labelled SA suggests that SA with an appropriate substituent at C-9 could be effectively taken up from the bloodstream by tumors.^5^ To circumvent potential recognition of α-2,3/6-sialosides by endogenous lectins,^9^ pNIR@P@SA with C-9 linked SA, an abiotic linkage potentially inert to lectins, was designed for *in vivo* tumor targeting.

**Fig. 2:**
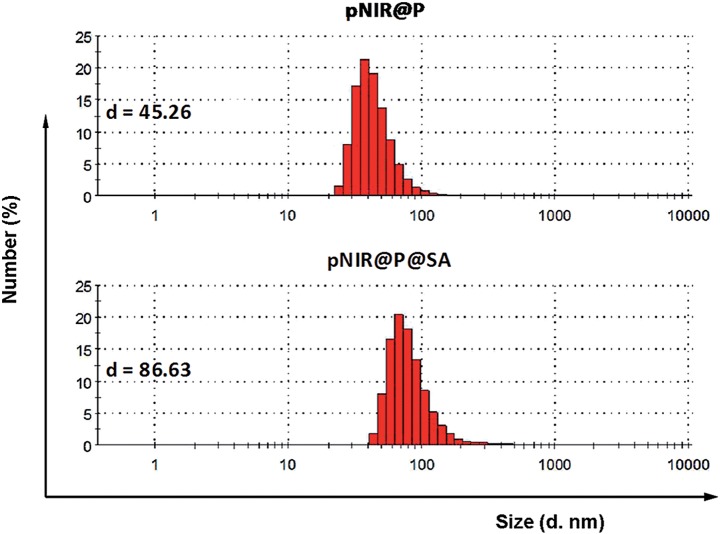
Diameters of pNIR@P@SA and pNIR@P measured by dynamic light scattering.

Apart from targetability, probes switched to signal-on state in tumors while remaining silent at off-target settings are advantageous for low background imaging.^10^ As such, rhodamine derivatives with intramolecular spiro rings have been employed for tumor detection by lysosomal acidity triggered fluorogenic opening of the rings.^11^ NIR dyes are superior to rhodamines for *in vivo* imaging owing to minimal autofluorescence of biological tissues in the NIR window.^12^ Hence pNIR, a pH responsive profluorophore with an intramolecular lactam,^5*b*^ was used as the lysosome acidity reporting element in this report. Poly[styrene-*alter*-(maleic acid)] is biocompatible, as its conjugate with neocarzinostatin has been clinically approved for liver cancer treatment.^13^ In addition, anionic poly[styrene-*alter*-(maleic acid)] derivatives exhibit low non-specific binding with mammalian cells due to Coulombic repulsion with negatively charged cell surface constituents.^11b,14^ As such, poly[styrene-*alter*-(maleic acid)]_40_, chosen as the carrier, was sequentially amidated with pNIR and 9-amino-9-deoxy-5-*N*-acetyl-neuraminic acid (9-NH_2_-SA) in dimethylformamide (Fig. 1). The resultant solution was hydrolyzed with aqueous sodium carbonate solution to abolish residual anhydride, dialyzed against distilled water, and then sonicated to afford nanoscopic pNIR@P@SA by self-assembly. Similarly, poly[styrene-*alter*-(maleic anhydride)]_40_ amidated with pNIR alone was prepared and used as the control (pNIR@P). Dynamic light scattering analysis shows that the statistical mean diameters are 86.63 nm and 45.26 nm for pNIR@P@SA and pNIR@P, respectively (Fig. 2), confirming formation of nanoscaled vesicles. The Zeta potentials have been determined to be –69.0 mv for pNIR@P@SA and –61.5 mv for pNIR@P (ESI, Fig. S1†), which is consistent with the anionic nature of these polymer vesicles.

### Acidic pH mediated fluorescence activation of pNIR@P@SA

**Fig. 3:**
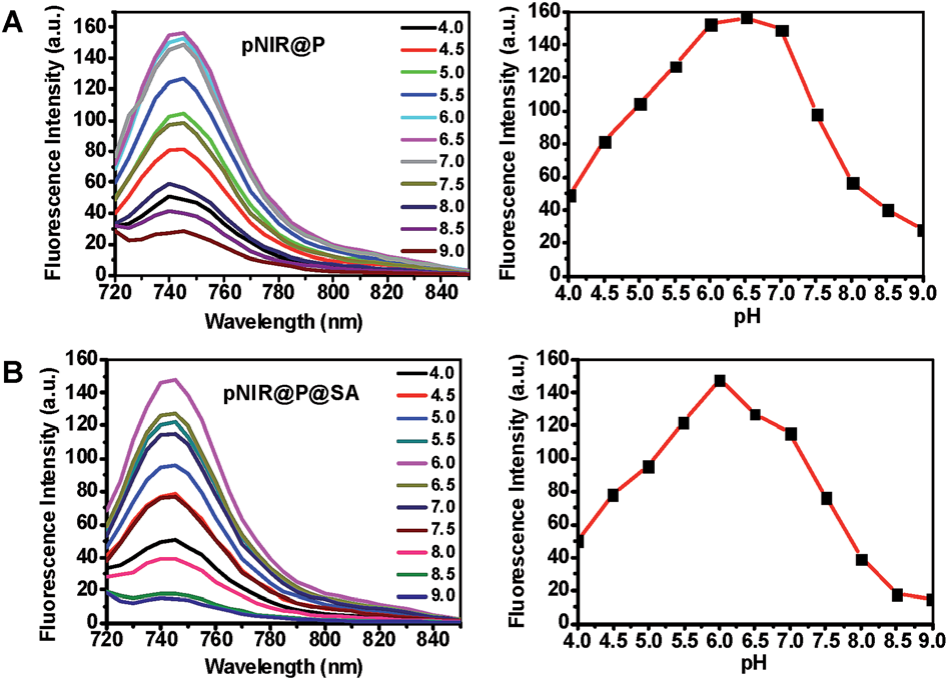
pH correlated fluorescence of pNIR@P (A) and pNIR@P@SA (B). The two polymersomes were spiked into sodium phosphate buffer (100 mM, pH 4.0–9.0) to a final concentration of 100 μg ml^–1^. Fluorescence emission of the solutions was recorded using *λ*_ex_@715 nm and fluorescence emission intensities@745 nm were plotted over buffer pH.

To ascertain pH responsiveness, pNIR@P@SA and pNIR@P were respectively spiked into a series of buffers of pH 4.0–9.0. The solutions were analyzed for UV-Vis-NIR absorption and fluorescence emission as a function of buffer pH. pNIR@P@SA and pNIR@P display dramatically enhanced fluorescence emission maxima at 745 nm in acidic buffer (pH 5.5–6.5) relative to alkaline buffer (Fig. 3). Absorption spectra show that both vesicles display absorbance peaks at 720 nm in acidic buffer which intensified as the buffer pH decreased (ESI, Fig. S2†). The spectral analysis validates the proton-triggered isomerization of pNIR into a NIR-absorbing species (Fig. 1), suggesting the applicability of pNIR-encapsulating vesicles for signal activation based illumination of endo-lysosomes (pH 4.0–6.5) in live cells.

### Illumination of lysosomes with pNIR@P@SA

**Fig. 4:**
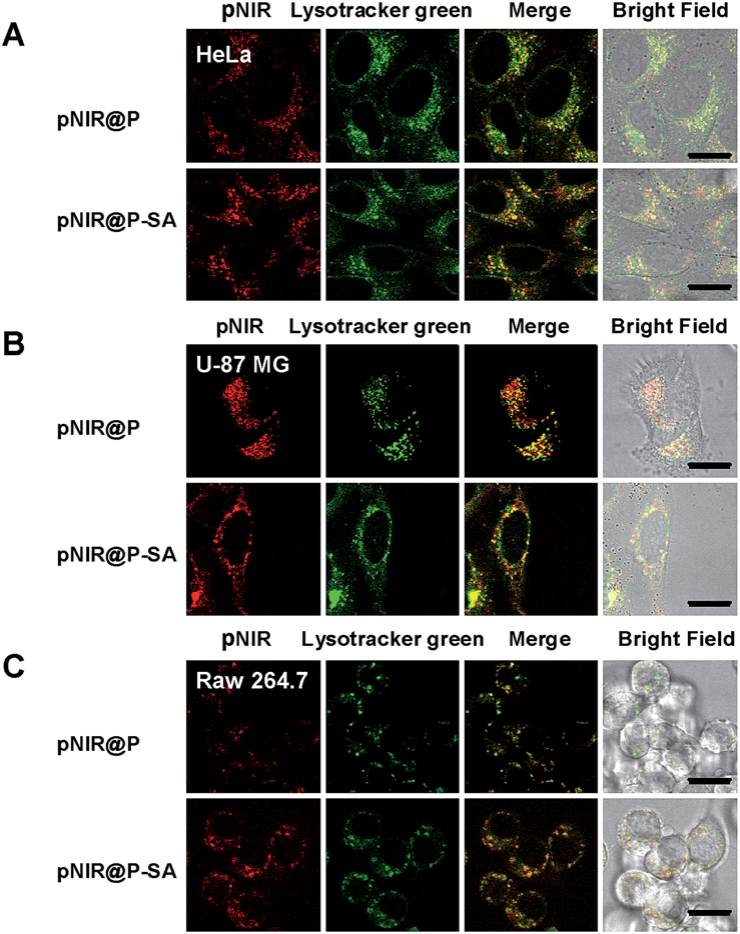
Illumination of lysosomes by pNIR@P@SA and pNIR@P. HeLa (A), U87-MG (B) and Raw 264.7 cells (C) were respectively cultured in DMEM spiked with pNIR@P@SA (100 μg ml^–1^) or pNIR@P (100 μg ml^–1^) for 1 h. The cells were stained with Lysotracker green (1 μM) in DMEM for 20 min, and then visualized using confocal fluorescence microscopy. Merging of the NIR signal (in red) and Lysotracker green (in green) demonstrates colocalization as indicated by the yellow areas. Bars, 10 μm.

Lysosomes are the major constituents of intracellular acidic compartments. We proceeded to investigate lysosome mediated activation of pNIR@P@SA in live cells. HeLa, U87-MG and Raw 264.7 cells were respectively cultured in Dulbecco's Modified Eagle's Medium (DMEM) supplemented with pNIR@P or pNIR@P@SA and then stained with LysoTracker Green DND-26 (referred to as Lysotracker green). As shown in Fig. 4, NIR signals were clearly observed in the three cell lines. Colocalization of NIR fluorescence with Lysotracker green, which is a lysosome-specific dye, reveals that pNIR@P and pNIR@P@SA could be taken up by mammalian cells from culture medium and then delivered into acidic lysosomes.

**Fig. 5:**
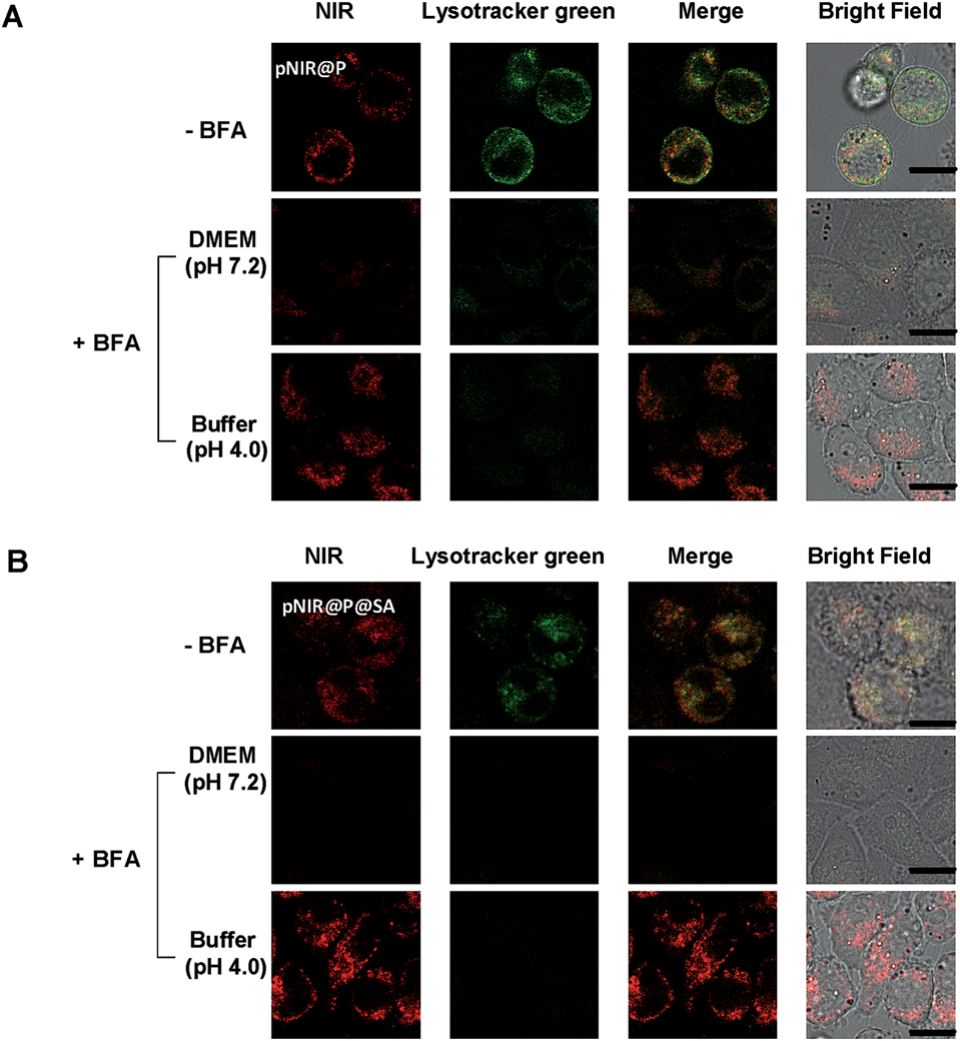
Acidity mediated “turn-on” fluorescence of pNIR@P (A) and pNIR@P@SA (B) in live cells. HeLa cells were cultured without or with BFA (100 nM) in DMEM for 4 h, incubated with 100 μg ml^–1^ of pNIR@P or pNIR@P@SA in DMEM for 1 h, and then stained with Lysotracker green (1 μM) in DMEM for 20 min. A portion of the BFA- and vesicle-loaded cells were resuspended in sodium phosphate buffer (pH 4, 100 mM) for 10 min. The cells were visualized using confocal fluorescence microscopy. The NIR signal is merged with Lysotracker green and the colocalization is indicated by yellow areas. Scale bars, 10 μm.

To substantiate lysosomal acidity-dependent activation, HeLa cells were first treated with bafilomycin A1 (BFA), and then co-stained with Lysotracker green and pNIR@P@SA or pNIR@P. BFA is a potent ATPase inhibitor and could effectively neutralize lysosomes.^15^ The lysosome-specific NIR signals largely vanish in BFA-treated cells (Fig. 5), indicating lysosomal acidity dependent signal activation of internalized vesicles. To ascertain the impact of BFA on vesicle uptake, BFA-treated HeLa cells were incubated with pNIR@P@SA or pNIR@P, and then resuspended in sodium phosphate buffer (pH 4.0) for 10 min. Confocal fluorescence microscopic images reveal the recovery of bright NIR signals within cells upon suspension in acidic media (Fig. 5), excluding hampered internalization of pNIR@P@SA and pNIR@P into BFA-treated cells. Collectively, these results confirm lysosomal acidity dependent fluorescence activation of endocytosed pNIR@P@SA in live cells. *In vitro* pH titration shows that pNIR@P@SA is strongly fluorescent in acidic media and yet moderately luminescent in a pH 7.2 buffer (Fig. 3). In contrast, pNIR@P@SA is nearly nonfluorescent in cytosolic pH (pH 7.2) in BFA-treated cells (Fig. 5), which is beneficial for the proposed lysosomal activation based tumor detection (Fig. 1).

### Fluorescence imaging of subcutaneous tumors in mice with pNIR@P@SA

**Fig. 6:**
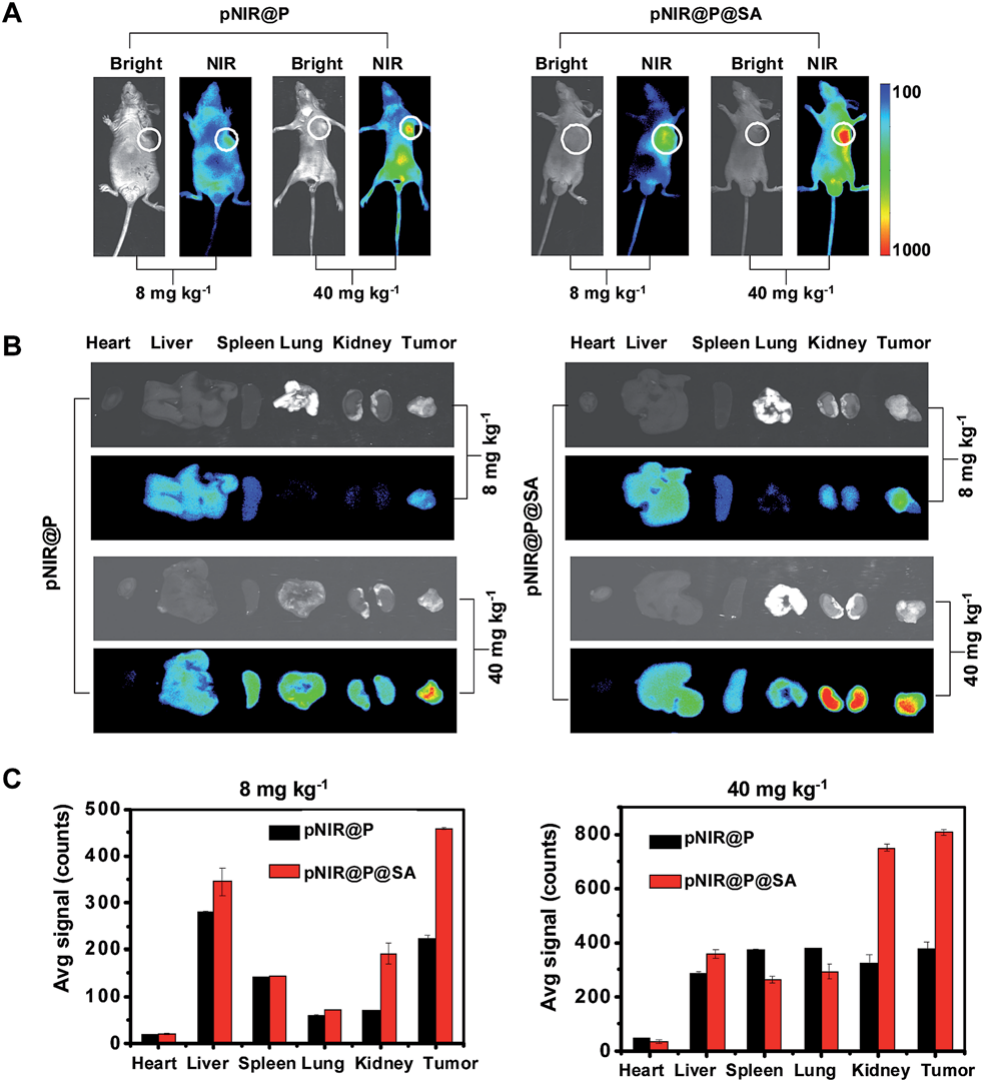
Superior illumination of subcutaneous tumors with pNIR@P@SA over pNIR@P. Nude mice with subcutaneous H22 tumors were intravenously injected with pNIR@P or pNIR@P@SA (8 mg kg^–1^ or 40 mg kg^–1^) *via* the tail vein. At 15 h after injection, the mice were imaged for whole body fluorescence (A). The tumor and selected organs harvested from the mice were imaged for *ex vivo* fluorescence (B). The bar graphs show tissue distributions of NIR fluorescence (C). The circles indicate the tumor location.

Shown to become fluorescent in lysosomes, pNIR@P@SA was evaluated for its efficacy to illuminate subcutaneous tumors in mice. Nude mice were subcutaneously inoculated with H22 hepatocellular carcinoma cells in the flank. The mice were maintained for 5–10 days after inoculation to allow development of H22 tumor xenografts. pNIR@P@SA and pNIR@P were respectively injected into the tumor-bearing mice *via* the tail vein. The mice were imaged for whole body fluorescence at 15 h following injection. Intense NIR signals are clearly identified in the subcutaneous tumors in mice treated with pNIR@P@SA whereas moderate NIR signals were detected in the subcutaneous tumors treated with pNIR@P at identical doses (Fig. 6A). The mice were sacrificed. The tumors and representative organs were excised and analyzed for *ex vivo* fluorescence emission. Consistent with the whole body imaging results (Fig. 6A), superior tumor-to-healthy organ fluorescence contrasts were identified in the tumors treated with pNIR@P@SA rather than in those treated with pNIR@P (Fig. 6B and C), validating the superior capacity of pNIR@P@SA to illuminate tumors *in vivo* and the beneficial role of the SA on the surface of the vesicles for enhanced tumor targeting efficiency.

**Fig. 7:**
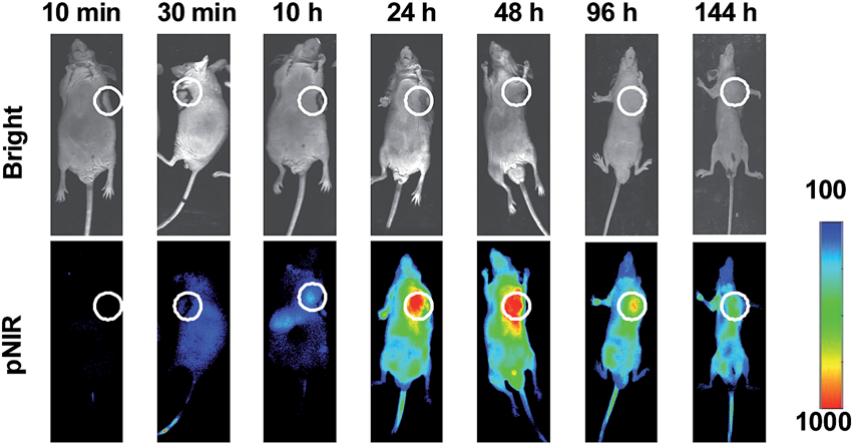
Time course for whole body fluorescence activation of pNIR@P@SA in tumor-bearing mice. Nude mice bearing H22 subcutaneous tumors were injected by tail vein with pNIR@P@SA (40 mg kg^–1^) and then monitored for whole body fluorescence at 10 min–144 h following vesicle injection. Circles indicate the location of subcutaneous tumors.

To probe the time course of *in vivo* activation of pNIR@P@SA, nude mice bearing subcutaneous H22-tumor xenografts were administered with pNIR@P@SA by tail vein and then monitored for whole body fluorescence at fixed time points. As shown in Fig. 7, NIR signals, negligible in mice up to 30 min after vesicle injection, reach a maxima in the tumor at 24–48 h and then attenuate at 96 h post injection. These results verify that pNIR@P@SA is nonfluorescent in the blood stream during circulation and then could be internalized and activated by tumors. The long-term retention of high tumor-to-background signal contrast is beneficial for extended practical tumor surgery. The dramatically decreased whole body NIR signals at 144 h post injection reveals effective *in vivo* clearance of pNIR@P@SA (Fig. 7), which is beneficial for *in vivo* biomedical application.

### High performance fluorescence imaging of liver tumor foci with pNIR@P@SA

**Fig. 8:**
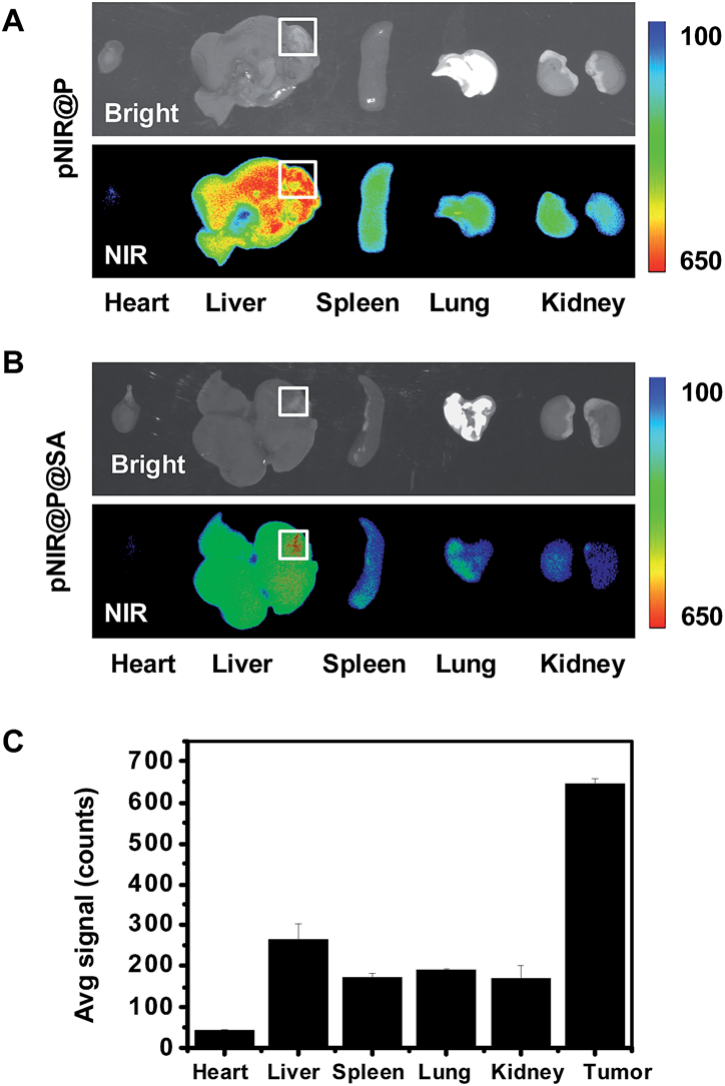
Illumination of tumor foci in liver with pNIR@P@SA. ICR mice with liver tumor xenografts were intravenously injected with 40 mg kg^–1^ of pNIR@P (A) or pNIR@P@SA (B), and then sacrificed 48 h post injection. The liver and selected organs were excised and probed for *ex vivo* fluorescence emission. The bar graph shows the fluorescence intensity of pNIR@P@SA in tumor foci, healthy liver tissue and other organs as indicated (C). Boxes indicate locations of liver tumor foci.

Hepatocellular carcinoma is a major health problem worldwide with 60 000 new cases diagnosed each year.^16^ Surgery combined with chemotherapy remains the primary choice for liver cancer therapy. As such, agents enabling precise detection of liver tumors are of clinical significance. pNIR@P@SA was evaluated for its capacity to illuminate tumor foci in liver. ICR mice with H22 hepatocellular carcinoma implants in the liver were injected with pNIR@P@SA or pNIR@P *via* the tail vein. At 48 h post injection, the mice were sacrificed. The tumor-bearing liver and other healthy organs were harvested and subjected to *ex vivo* fluorescence analysis. Intensive NIR signals were indiscriminately distributed in tumor foci and surrounding healthy liver tissue from mice treated with pNIR@P (Fig. 8A). In contrast, high fluorescence contrasts were identified in tumor foci over healthy liver tissue and organs from mice injected with pNIR@P@SA (Fig. 8B and C).

Hepatocytes efficiently capture and internalize nanoscale materials,^17^ but tumor targeting nanosystems with low levels of hepatic uptake remain challenging. In contrast with the indiscriminate intense fluorescence of pNIR@P in healthy liver tissue and tumor foci, the intense tumor-associated NIR signals and low levels of fluorescence in healthy liver portions further verify the beneficial impact of SA for *in vivo* tumor uptake (Fig. 8B). The obviously decreased fluorescence intensity of pNIR@P@SA over pNIR@P in the healthy portion of the liver at 48 h (Fig. 8B) post injection over that at 15 h (Fig. 6B) post injection shows that pNIR@P@SA displays long term retention in tumor foci whereas pNIR@P@SA in the healthy portion is poised for quick hepatic clearance. Although the cellular machinery or physiological factors responsible for tumor uptake of pNIR@P@SA remain to be elucidated, the results indicate the utility of SA as a tumor targeting ligand.

Symptoms of human hepatocellular carcinoma often occur until the tumors grow to 4–8 cm in diameter.^18^ A minimum of 1 cm clearance, known as minimal residual disease, is pursued by surgeons during cancer resection.^19^ As demonstrated in Fig. 8B, the size of the liver tumor discerned by pNIR@P@SA (∼4 mm) is significantly below minimal residual cancer (1 cm). The high tumor-to-healthy organ signal contrasts (Fig. 8C) and the capacity to distinguish millimeter-sized liver tumors shows the potential of SA as a tumor-targeting ligand in nanomedicine.

### Acid activatable photoacoustic property of pNIR@P@SA

Fluorescence imaging suffers from intense photon diffusion within soft tissues whereas acoustic imaging relies on the use of weakly scattered ultrasound and can image objects several centimeters deep in biological tissues.^20^ To date, optoacoustic bioimaging has been performed with the aid of exogenous contrast agents such as indocyanine green (ICG), conjugated polymers, and metallic nanoparticles.^21^

Distinct from ICG dye with an “always-on” optoacoustic signal, pNIR isomerizes in acidic media to give a NIR fluorophore with strong absorption at 650–750 nm (Fig. 3 and S2, ESI†). It is anticipated that a portion of the absorbed optical energy by pNIR in acidic media is released as fluorescence emission and heat, as the fluorescence quantum yield is <100%, suggesting the potential of pNIR as an acid activatable optoacoustic agent.

**Fig. 9:**
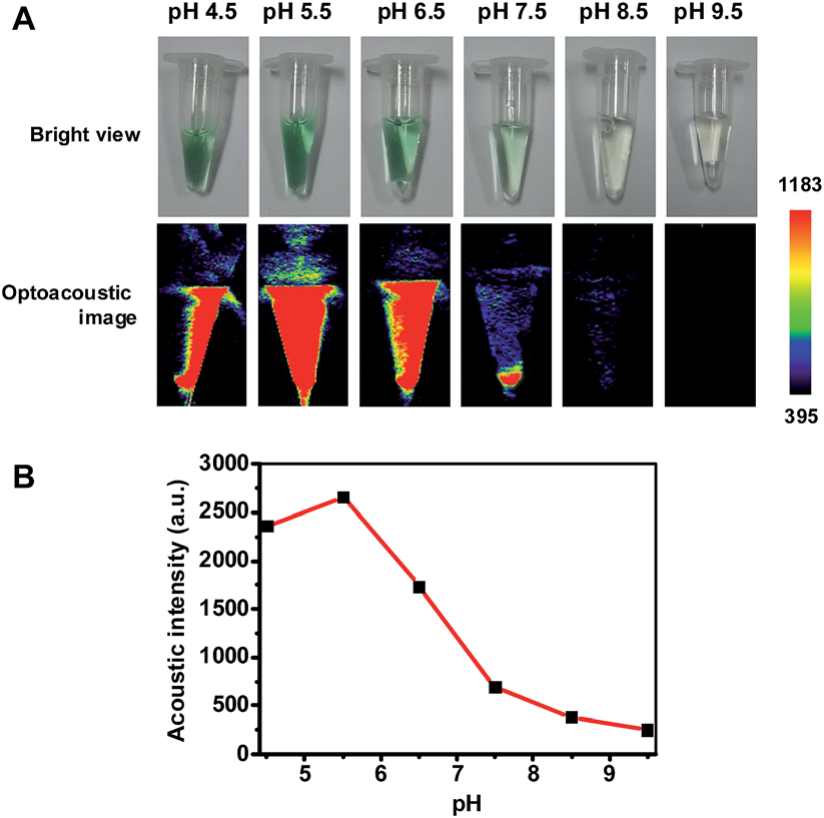
Acidic pH mediated activation of the photoacoustic property of pNIR@P@SA. pNIR@P@SA was spiked into sodium phosphate buffers (100 mM) of varying pH values pH 4.5–9.5 to a final concentration of 1 mg ml^–1^. Visual images and photoacoustic contrast for the solutions were recorded (A). The optoacoustic intensity was plotted over buffer pH (B).

Hence pNIR@P@SA was assessed for acid activatable photoacoustic tumor imaging. Ultrasound is generated from thermoelastic expansion caused by contrast agents excited by pulsed laser. To probe the photothermal effects, the solution containing pNIR@P@SA was exposed to 660 nm laser illumination at a power density of 0.5 W cm^–2^. Time course studies revealed a dramatically elevated temperature in the aforementioned solution over that of the probe-free solution (Fig. S3, ESI†), showing the capability of pNIR@P@SA to convert NIR irradiation into heat, proving its photothermal capability. Next, pNIR@P@SA was spiked into buffers with varying pH. The solutions were analyzed for photoacoustic intensity. As shown in Fig. 9, intense PA signals are observed in acidic buffers (pH 6.5–4.5) whereas weak or no signals were identified at neutral to alkaline conditions (pH 7.5–9.5). Since the SA moiety and the polymeric carrier remained structurally unchanged between pH 4–8, the acidity dependent “turn-on” optoacoustic contrast of pNIR@P@SA is clearly due to isomerization of pNIR into a fluorescent NIR moiety. To further corroborate this observation, pNIR and the control polymer (P@SA) were assayed for their pH dependent optoacoustic properties underlying optoacoustic imaging. It was shown that pNIR displays acid activatable photothermal effects whereas P@SA is inert under identical conditions (Fig. S4†). Taken together, these results validate acidic pH mediated “turn-on” optoacoustic signals of the pNIR moiety at pNIR@P@SA.

### Photoacoustic imaging of tumors in mice with pNIR@P@SA

**Fig. 10:**
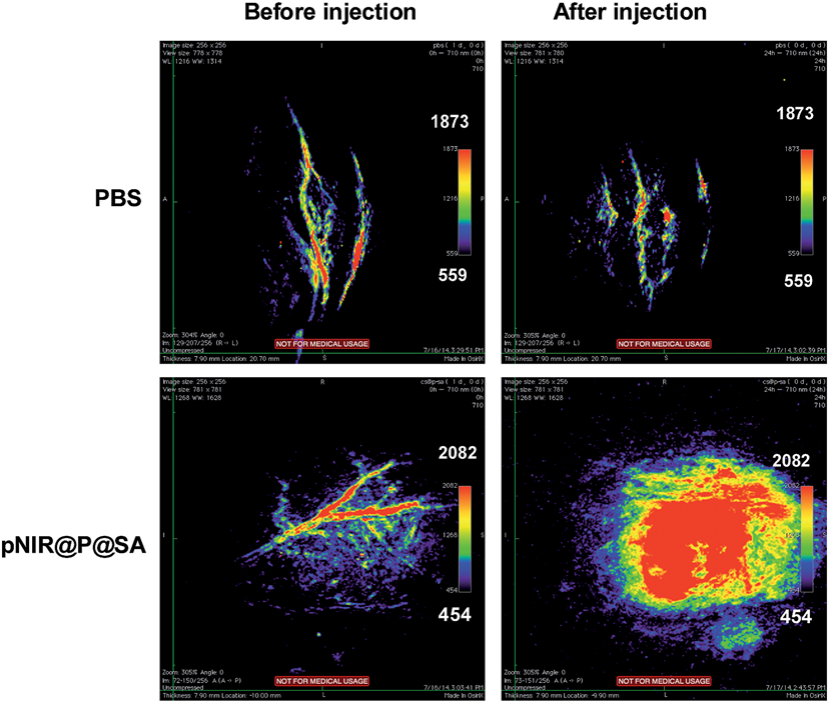
Photoacoustic tumor imaging in mice with pNIR@P@SA. Nude mice bearing H22 subcutaneous tumors were intravenously injected with PBS (100 μl) or pNIR@P@SA (40 mg kg^–1^). The mice were imaged 24 h after vesicle injection. Control images were obtained from mice before intravenous injection of pNIR@P@SA or PBS.

Nude mice bearing subcutaneous tumors were intravenously injected with pNIR@P@SA or phosphate buffered saline (PBS) and then probed for *in vivo* photoacoustic signals. As shown in Fig. 10, the intratumor vessels can be clearly visualized in the intact mice. This observation is consistent with reported optoacoustic imaging of blood vessels.^22^ Despite the background optoacoustic contrast resulting from endogenous biomolecules, dramatically increased optoacoustic signals are identified in the tumors from mice following tail vein injection with pNIR@P@SA whereas no variations in optoacoustic brightness are observed in subcutaneous tumors from mice treated with PBS (Fig. 10), proving the applicability of pNIR@P@SA for lysosomal acidity-activatable photoacoustic imaging of tumors. Albeit with limited tissue penetration, NIR fluorescence imaging has low background signals due to minimal biological autofluorescence in the NIR region (Fig. 7). Given the obvious optoacoustic contrast intrinsic of physiological constituents (*e.g.* blood vessels), pNIR@P@SA, with activatable fluorescence and photoacoustic signals, combines the advantages of both acoustic imaging (deep tissue penetration) and NIR fluorescence imaging (low background signals), which is of use for practical intraoperative tumor resection.

### Cytotoxicity of pNIR@P@SA

**Fig. 11:**
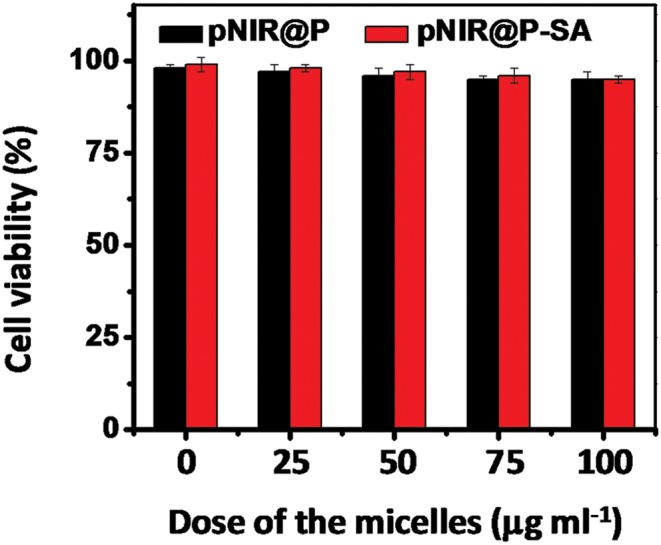
Cytotoxicity of pNIR@P@SA and pNIR@P. HeLa cells were cultured for 24 h in DMEM spiked with various amounts of pNIR@P or pNIR@P@SA (0, 25, 50, 100 μg ml^–1^). The cell number and cell viability were determined using trypan blue exclusion.

To probe the cytotoxicity of the nanovesicles, HeLa cells were cultured with varying levels of pNIR@P@SA or pNIR@P in DMEM. Cell viability was determined using a trypan blue exclusion test. No detrimental effects on cell viability were observed at doses up to 100 μg ml^–1^ after a 24 h incubation (Fig. 11), indicating that pNIR@P@SA have low cell toxicity. To ascertain systemic toxicity, pNIR@P@SA was intravenously injected into mice at doses of up to 160 mg kg^–1^ in healthy mice, which is 4 times higher than the doses employed for tumor imaging. The mice were regularly monitored for whole body fluorescence emission and adverse physiological effects after vesicle injection. Whole body fluorescence images of the mice show that NIR signals, maximal at 4 h post injection, dramatically decrease over time (Fig. S5, ESI†). The extremely low levels of NIR signals at 7 days post injection show high efficiency clearance of injected pNIR@P@SA. In addition, no signs of pain or fatigue could be observed in mice up to 7 days after nanovesicle administration. Poly[styrene-*alter*-(maleic acid)] is biocompatible and has been used as the carrier of neocarzinostatin for clinical treatment of primary hepatoma and secondary liver cancer in Japan.^13^ Consistently, our results shows that pNIR@P@SA is of low cytotoxicity and systemic toxicity, which are critical for *in vivo* imaging studies.

## Conclusion

We demonstrate the use of a multifunctional nanovesicle for signal activation based fluorescence and photoacoustic tumor imaging in mice. The nanovesicle, pNIR@P@SA, consists of surface-anchored sialic acid for tumor targeting, a biocompatible carrier of poly[styrene-*alter*-(maleic acid)], and a core of near infrared profluorophore poised for proton triggered isomerization to give NIR fluorescence and optoacoustic signals in lysosomes. pNIR@P@SA effectively illuminates subcutaneous tumor and millimeter-sized tumor foci in liver with high target-to-healthy organ signal contrasts, validating the potential of sialic acid as a tumor targeting ligand in nanomedicine. The distinguished tumor-associated fluorescence and acoustic contrasts demonstrate the applicability of pNIR@P@SA for dual modality tumor imaging. Integrating the advantages of NIR fluorescence (low background) and optoacoustic imaging (deep tissue penetration), this activatable nanosystem, readily modulated for imaging of different tumors by incorporation of cognate targeting entities on the vesicle surface, would be of broad interest for dual modality cancer diagnosis and imaging guided tumor surgery.
