# Supplementary material for: A carbohydrate-grafted nanovesicle with activatable optical and acoustic contrasts for dual modality high performance tumor imaging
Source: Chem Sci. 2014 Dec 22;6(3):2002–9. doi: 10.1039/c4sc03641g (PMC5496387; doi:10.1039/c4sc03641g)
Supplement: Supplementary file 1 [file SC-006-C4SC03641G-s001.pdf]

Electronic Supplementary Material (ESI) for Chemical Science.
This journal is © The Royal Society of Chemistry 2014

Supplementary materials

A carbohydrate-grafted nanovesicle with activatable optical and acoustic contrasts for dual modality high performance tumor imaging

Xuanjun Wu,[a] Bijuan Lin,[a] Mingzhu Yu,[a] Liu Yang,[a] Jiahuai Han,[b] and Shoufa Han[a,*]

[a]Department of Chemical Biology, College of Chemistry and Chemical Engineering, the Key Laboratory for Chemical Biology of Fujian Province, The MOE Key Laboratory of Spectrochemical Analysis & Instrumentation, and Innovation Center for Cell Biology, Xiamen University; [b]State key Laboratory of Cellular Stress Biology, Innovation Center for Cell Biology, School of Life Sciences, Xiamen University, Xiamen, 361005, China; Tel: 86-0592-2181728; E-mail: shoufa@xmu.edu.cn

Experimental

Material and methods

LysoTracker Green DND-26 was purchased from Invitrogen. Dulbecco's Modified Eagle Medium (DMEM) and GIBCO fetal bovine serum (FBS) were purchased from Sigma-aldrich. FBS was heat-inactivated at 56 °C for 30 min before use. pNIR was synthesized according to a reported procedure.[1] 9-Amino-9-deoxy-5-N-acetylneuraminic acid was prepared following a reported literature.[2] Poly[styrene-alt-(maleic anhydride)]$_{40}$ were available from previous study.[3] All other chemicals were used as received from Alfa Aesar. Distilled water was used for the preparation of all aqueous solution. Hela cells, U-87 MG and Raw 264.7 cells were obtained from American Type Culture Collection and grown at 37 °C under 5% CO_2 in DMEM.

The fluorescence spectra and UV-vis absorption spectra were recorded on a spectrofluorimeter (Spectramax M5, Molecular Device). Dynamic light scattering and Zeta potential analysis of the micelles were performed on Zetasizer Nano ZS (ZEN3500, Malvern). Confocal microscopic images were obtained on Leica SP5 using the following filters: λex@488 nm and λem@500-530 nm for Lysotracker green; λex@633 nm and λem@700-790 nm for pNIR. Fluorescence images were merged using Photoshop CS 6.0. Graph by origin 8.5 software. Fluorescence quantification of organs of mice were performed on Carestream FX PRO *in vivo* imaging system using an excitation filter of 690 nm and an emission filter of 750 nm. Data were analyzed with Carestream MI SE. Nude mice and ICR mice were gifts from Xiamen University Laboratory Animal Center. H22 hepatocellular carcinoma cells were collected from peritoneal cavity of tumor-bearing mice and then used for transplantation of tumors in mice. Photoacoustic imaging was performed on a 3-D high-resolution small animal imaging system (Endra Nexus 128, America) using NIR laser of 710 nm. All animal experiments were performed in accordance with the guidelines of Xiamen University's Animal Care and Use Committee.

Preparation and characterization of pNIR@P@SA and pNIR@P

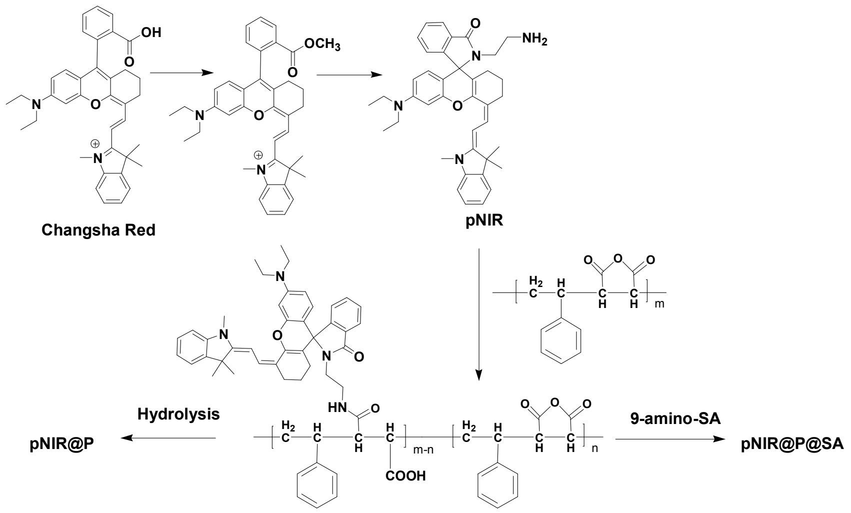

Scheme S1. Synthesis of pNIR and pNIR conjugated polymeric micelles.

Changsha Red was syntheszied following a published procedure.[4] pNIR was prepared following a reported procedure.[1] Briefly, Changsha Red (5 g) was added to a flask containing methanol (100 ml). To the solution in ice bath was added thionyl chloride (10 ml) dropwise. The resultant solution was heated at 70℃ for 12 h. The solution was concentrated by rotary evaporation to remove the solvent and and the resulting residue was dissolved in methanol (20 ml). To the solution was added ethylenediamine (10 ml). The reaction solution was heated at 70℃ for 1 h and then concentrated by evaporation. The residue was purified on silica gel chromatography using dichloromethane/hexanes/triethylamine (10:10:1) as the eluent to give pNIR as a pale yellow solid in 36% yield. The analytical data of pNIR are identical to reported values.[1] ^{1}H-NMR (400 MHz, DMSO-d_6): δ 7.74 (d, 1H, J = 6.96), 7.53-7.40 (m, 3H), 7.24 (d, 1H, J = 7.04), 7.12 (t, 2H, J = 7.56), 6.78 (q, 2H, J = 5.04), 6.37-6.21 (m, 3H), 5.39 (d, 1H, J = 12.68), 3.31 (q, 4H, J = 6.96), 3.13 (s, 3H), 3.10-3.02 (m, 2H), 2.60 (m, 1H), 2.50-2.43 (m, 1H), 1.63 (d, 6H, J = 3.56), 1.57-1.48 (m, 3H), 1.37-1.18 (m, 3H), 1.09 (t, 6H, J = 7.00); ^{13}C-NMR (100 MHz, DMSO-d_6): 167.72, 157.75, 152.48, 151.65, 148.65, 147.56, 145.41, 138.61, 132.82, 131.88, 128.77, 128.59, 128.14, 123.67, 122.66, 122.00, 119.75, 119.70, 108.81, 106.56, 105.28, 103.90, 97.26, 92.41, 66.21, 55.34, 45.34, 44.10, 43.53, 29.33, 28.54, 28.43, 25.20, 12.19.

To a flask containing anhydrous dimethylformamide (DMF, 10 ml) was added poly[(styrene-*alter*-(maleic anhydride)] (700 mg, mw: 10, 000) in the presence of triethylamine (1 ml). The solution of pNIR (100 mg) in anhydrous DMF (1 ml) was dropwise added and the reaction was stirred for 2 h. After that, the solution was divided into 2 equal portions to which was respectively added 9-Amino-9-deoxy-5-N-acetylneuraminic acid (9-Amino-SA, 175 mg) or no addition. The mixtures were stirred at rt for overnight followed by addition of aqueous Na_2CO_3 solution (1 M, 5 ml). The mixtures were first stirred at rt for 1 h and then extensively dialyzed against distilled water using a dialysis tube (MWCO 3500) to remove excess reagents and DMF. The solutions were respectively lyophilized and the resultant solids were dissolved in distilled water and then ultrasonicated for 30 min to afford pNIR@P@SA or pNIR@P. The aqueous solutions of these micelles (1 mg ml^{-1}) were respectively characterized by Zetasizer Nano ZS for their hydrodynamic sizes and zeta potentials. The aqueous solutions of these micelles (10 mg ml^{-1}) were used for the in *vivo* experiment. In parallel experiment, to a flask containing anhydrous dimethylformamide (DMF, 10 ml) was added poly[(styrene-*alter*-(maleic anhydride)] (700 mg, mw: 10, 000) in the presence of triethylamine (1 ml). and then 9-Amino-SA (175 mg). The mixtures were stirred at rt for overnight followed by addition of aqueous Na_2CO_3 solution (1 M, 5 ml). The mixtures were first stirred at rt for 1 h and then extensively dialyzed against distilled water using a dialysis tube (MWCO 3500) to remove excess reagents and DMF. The solutions were respectively lyophilized and the resultant solids were dissolved in distilled water and then ultrasonicated for 30 min to afford P@SA.

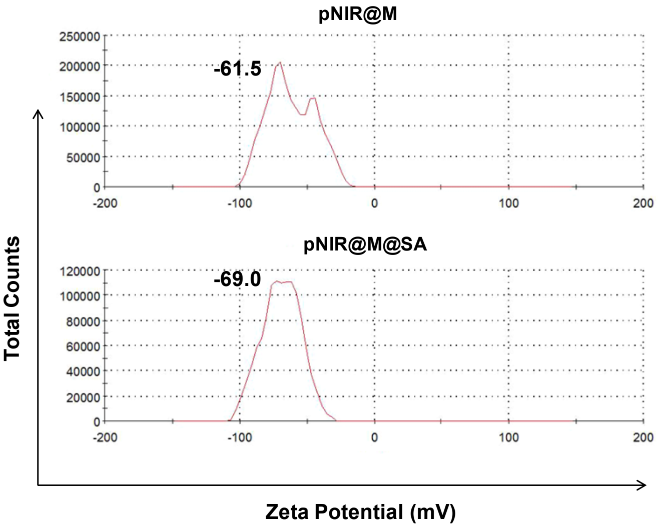

Fig. S1 Zeta potentials of pNIR@P@SA and pNIR@P. pNIR@P@SA and pNIR@P were respectively spiked into distilled water to a final concentration of 1 mg ml^{-1}. The solutions were analyzed by dynamic light scattering.

pH titration of pNIR@P@SA and pNIR@P

Aliquots of stock solution of (10 µl, 10 mg ml^{-1} in distilled water) or @SA (10 µl, 10 mg ml^{-1} in distilled water) were respectively added to sodium phosphate buffers (100 mM, 1 ml) of various pH containing 10 % acetonitrile (V/V). The fluorescence emission of the solutions was recorded as a function of buffer pH using *λex*@715 nm. The titration curves were plotted by fluorescence emission intensities@745 nm versus pH. UV-vis-NIR absorption of the solutions was recorded as a function of buffer pH. The titration curves were plotted by absorbance@720 nm versus buffer pH.

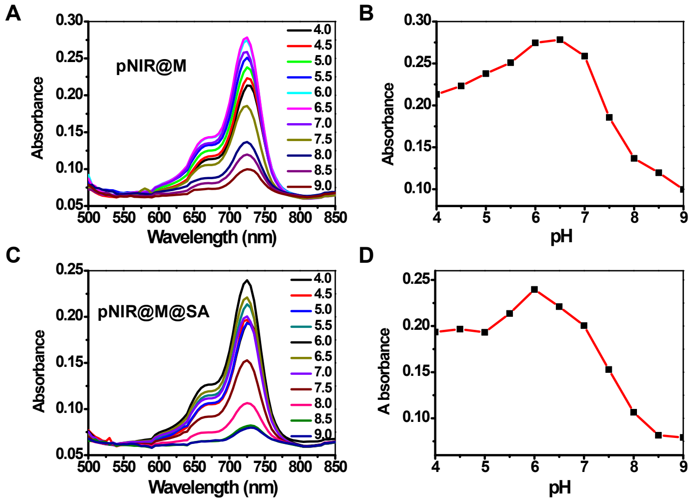

Fig. S2 pH depednent optical absorption of pNIR@P@SA and pNIR@P. pNIR@P and pNIR@P@SA were respectively spiked into sodium phosphate buffer (100 mM, pH 4.0-9.0) containing 10% acetonitrile (v/v) to a final concentration of 100 µg ml^{-1}. Absorption spectra of the solutions were recorded. The absorbance at 720 nm was plotted over buffer pH.

Illumination of lysosomes with pNIR@P@SA and pNIR@P

Hela cells, U-87 MG cells and Raw 264.7 cells were seeded on 35 mm glass-bottom dishes (NEST) and incubated for 24 h in DMEM supplemented with 10% FBS. The cells were spiked with pNIR@P@SA or for 1 h in DMEM and washed with PBS (1 ml) and further incubated with Lysotracker green (1 µM) for 20 min in DMEM. The resultant cells were placed in fresh medium and

then analyzed by confocal fluorescence microscopy.

For BFA-treated: HeLa cells pre-treated without or with BFA (100 nM) in DMEM for 4 h were spiked with pNIR@P (100 μg ml^{-1}) or pNIR@P@SA (100 μg ml^{-1}) for 1 h in DMEM supplemented with 10% FBS and washed with PBS (1 ml) and then stained with Lysotracker green (1 μM) in DMEM for 20 min. The cells incubated with both BFA and the polymersomes were further resuspended in sodium phosphate buffer (pH 4, 100 mM) for 10 min. The resultant cells were visualized with a confocal fluorescence microscope.

Cytotoxicity of pNIR@P@SA and pNIR@P

HeLa cells were cultured in DMEM containing various levels (0, 25, 50, 75, 100 μg ml^{-1}) of pNIR@P@SA or pNIR@P for 24 h at 37 ℃ with 5% CO_2. The cells were stained with trypan blue. Cell number and cell viability were determined using the trypan blue exclusion test.

Fluorescence imaging of subcutaneous tumors in mice with pNIR@P@SA

Nude mice were xenografted by subcutaneous injections of H22 cells ($1x10^6$). At 5-10 days after the transplantation, a cohort of tumor-bearing mice were injected intravenously via the tail vein with pNIR@P@SA or pNIR@P (8 mg kg^{-1} or 40 mg kg^{-1}). At 15 h post-injection, whole body and disected organs of the mice were imaged by *ex vivo* analysis for NIR fluorescence intensity.

Time course of fluorescence imaging of subcutaneous tumors in mice with pNIR@P@SA

Nude mice were xenografted by subcutaneous injections of H22 cells ($1x10^6$). At 5-10 days after the transplantation, a cohort of tumor-bearing mice were injected intravenously via tail vein with pNIR@P@SA or pNIR@P (40 mg kg^{-1}). At 30 min-144 h post-injection, whole bodies of the mice were imaged by *ex vivo* analysis for NIR fluorescence intensity.

Fluorescece detection of liver tumors in mice with pNIR@P@SA

ICR mice xenografted in the liver with H22 cells were obtained from the animal center of Xiamen University. At 5-10 days after transplantation, the mice were injected intravenously via the tail vein with pNIR@P@SA or pNIR@P (40 mg kg^{-1} in mice). After 48 h, the mice were anesthetized. The liver and representative healthy organs were excised, washed with PBS and then subjected to *ex vivo* analysis for NIR fluorescence intensity.

Photothermal effects of pNIR@P@SA

Sodium phosphate buffer (100 mM, pH 4.5) was spiked with or without pNIR@P@SA to a final concentration of 0.1 mg ml^{-1}. The solutions were irradiated with NIR laser for 10 min (660 nm, 0.5 W cm^{-2}) and the temperature of the solutions was recorded over irradiation time.

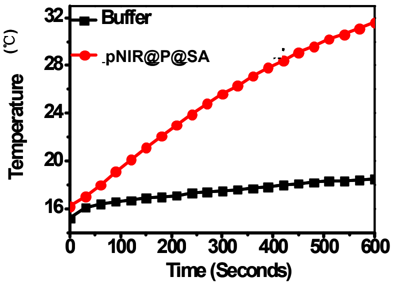

Fig. S3 Photothermal effects of pNIR@P@SA. The temperature of sodium phosphate buffer (100 mM, pH 4.5) containing 10% acetonitrile (v/v) with pNIR@P@SA (0 or 1 mg ml^{-1}) was recorded over the

time of irradiation with NIR laser (660 nm, 0.5 W cm^{-2}).

In an separate assays, sodium phosphate buffer (100 mM, pH 4.5) was respectively spiked with or without P@SA (1 mg ml^{-1}) and pNIR (0.1 mg ml^{-1}). The solutions were irradiated with NIR laser for 10 min (660 nm, 0.5 W cm^{-2}) and the temperature of the solutions was recorded over irradiation time.

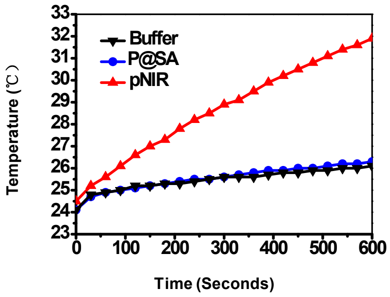

Fig. S4 Photothermal effects of pNIR. The temperature of sodium phosphate buffer (100 mM, pH 4.5) containing 10% acetonitrile (v/v) with P@SA (0 or 1 mg ml^{-1}) or pNIR (0.1 mg ml^{-1}) was recorded over the time of irradiation with NIR laser (660 nm, 0.5 W cm^{-2}).

pH titration of optoacoustic response of pNIR@P@SA

pNIR@P@SA was spiked into a serial of sodium phosphate buffer (100 mM, pH 4.5, 5.5, 6.5, 7.5, 8.5 and 9.5) to a final concentration of 1 mg ml^{-1}. The solutions were imaged for optoacoustic signal intensity.

Photoacoustic imaging of subcutaneous tumors with pNIR@P@SA

Nude mice were xenografted by subcutaneous injections of H22 cells ($1x10^{6}$). At 5-10 days after the transplantation, a cohort of tumor-bearing mice were injected intravenously via the tail vein with pNIR@P@SA (40 mg kg^{-1}) or PBS (100 µl). The mice were imaged for photoacoustic signals before and 24 h post-injection.

Cytotoxicity of pNIR@P@SA

For cell toxicity: HeLa cells were respectively cultured for 24 h in DMEM medium containing various levels of pNIR@P@SA (0-100 µg ml^{-1}) or pNIR@P (0-100 µg ml^{-1}). The cell number and cell viability were determined by trypan blue exclusion assay.

For systemic toxicity: A healthy mice was intravenously injected with pNIR@P@SA (160 mg kg^{-1}) via tail vein. The mice was monitored regularly for whole body NIR fluorescecne and adverse physiological effects. At 7 days post-injection, the mice was sacrificed and the tumor and selected organ was harvested and examined for *ex vivo* NIR fluorescence.

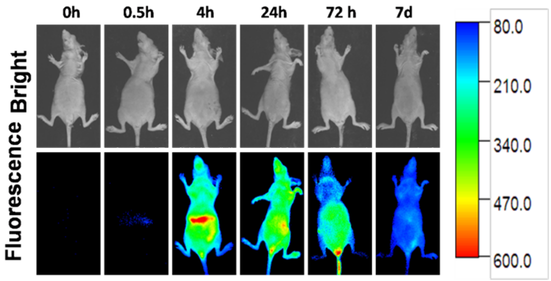

Fig. S5 Whole body imaging with over-dosed pNIR@P@SA. ICR mice was intravenously injected with pNIR@P@SA (160 mg kg^{-1}) for 7 days *via* tail vein and imaged for *in vivo* NIR fluorescence emission.

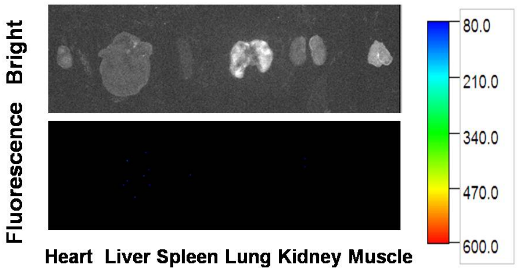

Fig. S6 Biodistribution of over-dosed in health mice. ICR mice was intravenously injected with pNIR@P@SA (160 mg kg^{-1}) and then sacrificed 7 days postinjection. The representative organs were dissected and analyzed for *ex vivo* fluorescence emission.

References

1. Wu, X. et al. A sialic acid-targeted near-infrared theranostic for signal activation based intraoperative tumor ablation *Chem Sci*, in press (2014).
2. Han, S., Collins, B.E., Bengtson, P. & Paulson, J.C. Homomultimeric complexes of CD22 in B cells revealed by protein-glycan cross-linking. *Nat Chem Biol* **1**, 93-97 (2005).
3. Li, Z. et al. Rhodamine-deoxylactam functionalized poly[styrene-alter-(maleic acid)]s as lysosome activatable probes for intraoperative detection of tumors *Chem Sci* **3**, 2941-2948 (2012).
4. Yuan, L., Lin, W., Yang, Y. & Chen, H. A unique class of near-infrared functional fluorescent dyes with carboxylic-acid-modulated fluorescence ON/OFF switching: rational design, synthesis, optical properties, theoretical calculations, and applications for fluorescence imaging in living animals. *J Am Chem Soc* **134**, 1200-1211 (2012).
